# Changes in dietary intake, chronotype and sleep pattern upon Ramadan among healthy adults in Jeddah, Saudi Arabia: A prospective study

**DOI:** 10.3389/fnut.2022.966861

**Published:** 2022-09-02

**Authors:** Ameera Alzhrani, Maha H. Alhussain, Ahmed S. BaHammam

**Affiliations:** ^1^Department of Food Science and Nutrition, College of Food and Agriculture Sciences, King Saud University, Riyadh, Saudi Arabia; ^2^Department of Medicine, The University Sleep Disorders Center, College of Medicine, King Saud University, Riyadh, Saudi Arabia; ^3^Strategic Technologies Program of the National Plan for Sciences and Technology and Innovation in the Kingdom of Saudi Arabia (08-MED511-02), Riyadh, Saudi Arabia

**Keywords:** intermittent fasting, energy intake, circadian rhythm, sleep, BMI

## Abstract

**Background:**

Notable lifestyle changes can occur in Ramadan due to the sudden shift in eating routine with fasting during daylight hours. This study aimed to examine the changes in dietary intakes, chronotype, sleep pattern, and physical activity level before and during Ramadan in healthy adults.

**Methods:**

This study was conducted in Jeddah city, Saudi Arabia, and convenience sampling was used. To compare dietary, chronotype and sleep pattern changes before and during Ramadan, data were collected in two separate periods: the first period was 2 months before Ramadan, and the second period was during the last 3 weeks of Ramadan. Dietary intake was assessed using 24-h food recall and chronotype using Morningness-Eveningness Questionnaire. Daytime sleepiness and sleep duration were assessed using the Epworth sleepiness scale and a sleep diary for seven consecutive days, respectively. Anthropometric measurements were also taken across the study periods.

**Results:**

A total of 115 adults (96 females and 19 males) were included in the study. Significant increases in daily calorie and carbohydrate intakes during Ramadan than before Ramadan were noted (calorie intake: 1,482.9 ± 536.4 kcal/day before Ramdan vs. 1,635.5 ± 635.1 kcal/day during Ramadan; carbohydrate intake: 180.8 ± 72.1 g/day before Ramadan vs. 202.6 ± 88.7 g/day during Ramadan; *p* < 0.05). Chronotypes and daytime sleepiness were also associated significantly with Ramadan fasting. A significant slight reduction in body weight during Ramadan was observed (66.4 ± 18.1 kg before Ramdan vs. 66.1 ± 17.8 kg before Ramadan and during Ramadan, respectively; *p* < 0.05).

**Conclusion:**

This study indicates that Ramadan diurnal fasting was associated with greater calorie and carbohydrate intake, changes in chronotype, and daytime sleepiness. The study also suggests that Ramadan diurnal fasting model may be a promising weight loss strategy.

## Introduction

Intermittent fasting has been suggested as an appropriate approach that can ameliorate many lifestyle-related complications, including chronic diseases (1). Since Ramadan diurnal fasting is a model of intermittent fasting, its effect on health is of contemporary interest. Furthermore, accumulating evidence has supported the health-related benefit of Ramadan diurnal fasting (2–4).

During Ramadan, healthy adult Muslims abstain from eating and drinking from dawn to sunset; this annual practice is the fourth pillar of the five pillars of Islam. Profound changes in daily lifestyle routine, including dietary intakes, sleep patterns, and physical activity, are observed during Ramadan, compared with other months of the year among Islamic societies (5, 6). The eating period in Ramadan is switched to nighttime, resulting in a delay in meal timing and frequency. Fast performers are expected to eat two main meals, breakfast at sunset and suhur (a pre-dawn meal) nearly 30 min before dawn, and to get a good night's sleep (7). This practice is anticipated to cause weight loss and improved physiological factors related to increased weight (7). However, this is not always the case in real life, as several lifestyle and cultural factors intermingle with the fasting practice during Ramadan (7, 8). For example, there is a tendency to consume a greater variety of foods and more sugary or high-calorie beverages during Ramadan than during other months (9, 10). Hypothetically, eating meals at unusual times (changing mealtimes) can affect circadian rhythm and disturb standard sleep patterns (4). In addition, recent evidence indicates that consuming high caloric food at the wrong time of day may result in misalignment between central and peripheral body clocks and may consequently impair metabolism and lead to weight gain (11–13).

Conflicting data have been reported regarding the daily intake of calories and macronutrients during Ramadan (14). For example, a study conducted in Turkey found that the total calorie, carbohydrate, and protein intake were significantly reduced during Ramadan compared with before Ramadan (15). However, there are cultural differences between different Muslim countries, and no study has assessed dietary changes during Ramadan in Saudi Arabia (16).

Changes in mealtime may also affect sleep patterns. A recent systematic review and meta-analysis reported a decrease in total sleep duration during Ramadan and an increase in the Epworth sleepiness scale (ESS) score compared with before Ramadan (17).

Previous studies examining the effects of Ramadan diurnal fasting on physical activity level (PAL) have reported conflicting results, and most of them included men (4). A Malaysian study demonstrated no significant differences in PAL before, during, and after Ramadan (18). On the other hand, Eltoum et al. (19), reported a significant decrease in PAL from 76% before Ramadan to 44% during Ramadan among the Saudi population. The present study aimed to investigate the changes in dietary intake, chronotype, sleep patterns, and PAL before and during Ramadan among healthy adults in Saudi Arabia.

## Materials and methods

### Study population and ethics

Healthy adults aged between 18 and 44 years (mean ± SD: 29.6 ± 6.6 years) from the general public in Jeddah city, Saudi Arabia, participated in this study *via* a poster advertisement sent to Community Development Commissions of Jeddah city and *via* social media snowball recruitment.

The study participants' inclusion criteria were as follows: adults who self-reported themselves as being healthy, age between 18 and 45 years, BMI < 30 kg/m^2^, not under medications other than oral contraceptives, not pregnant or lactating women, not dieting, and stable weight during the past 3 months.

The study was conducted according to the principles of the Declaration of Helsinki. Ethical approval was obtained from Institutional Review Board (IRB), College of Medicine, King Saud University (approval No. E-19-3706). Before being enrolled in the study, all participants were briefed on study aspects and were allowed to ask any questions. Informed consent was obtained from all participants.

### Study protocol

This repeated measures study was conducted over 3 months for two separate periods: the first period was the 2 months before Ramadan (from Rajab to 28 Sha'aban 1440 Hijri; corresponding to 8 March to 3 May 2019), and the second period was during Ramadan (from 8 to 27 Ramadan 1440 Hijri; corresponding to 13 May to 1 June 2019). Fasting was ~15 h from dawn (4:20 a.m) until sunset (7 p.m). During each period, participants were asked to visit the Community Development Commissions to collect the data, including anthropometry measurements, dietary intake, sleep pattern, and physical activity.

### Anthropometric measurements

During each of the two periods, the same investigator assessed anthropometric measurements according to standard protocol (20). Height was measured in centimeters (cm) to the nearest 0.1 cm. Body weight was measured in kilograms (kg) to the nearest 0.1 kg using a digital body composition monitor (Omron BF511, OMRON Healthcare Co., Kyoto, Japan). Body fat percentage and visceral fat were also obtained using Omron device (21). BMI was calculated using the standard formula: weight (kg)/height (m^2^).

### Dietary intake

A 24-h food recall was taken from each participant during the two periods to assess their dietary intake. Detailed instructions on how to complete the food recall were provided by trained staff to participants using household measures (22). Intakes of calories and macronutrients (carbohydrates, proteins, fats) were assessed using the Food Processor software (version 7.60 ESHA Research, Inc, Salem, OR/USA) (23). A trained dietitian reviewed the data, and exclusions were made for those reporting calories at the extreme low and high ends of reported energy intake (fewer than an average of 500 and >3,500 kcal per day for women, and fewer than an average of 800 and >4,200 kcal per day for men (24).

A self-administrated questionnaire was also used to collect further data regarding participants' dietary habits. The questionnaire consisted of questions about the number and type of meals and snacks, meal-timing, coffee, and tea consumption. Validation of the questionnaire was performed by two academic professors specialized in nutrition, and ambiguous questions were either eliminated or revised based on their feedback.

### Chronotypes

The term “chronotype” is utilized to refer to personal variations in sleep-wake patterns. Individuals who sleep early, wake up early, and think and function better in the morning are categorized as morning types; on the other hand, persons who sleep late, get up late, and perform better in the afternoon are categorized as evening types (25). The Morningness-Eveningness Questionnaire (MEQr), a self-evaluation questionnaire worked out by Horne and Östberg in 1976 (26), has successfully identified human circadian typology in experimental and applied research (27). Three questions determine the desired morning rise time, evening bedtime, and the hour of the day when individual efficiency is maximum. The two other questions assess the level of fatigue within the first half-hour after rising in the morning and the circadian type to which the respondent deems himself/herself to fit.

The validated Arabic version of the MEQr-short form, was used to determine chronotypes for participants during the two periods of the study (27, 28). The MEQr questions are multiple-choice, with each answer assigned a specific score. The final sum of these scores results in an overall score ranging between 4 and 25. Higher scores (18–25) indicate morning-type. The lower scores (4–11) indicate evening-types (E-types). The intermediate score (12–17) indicates participants classified as neutral types, i.e., intermediate characteristics between morning and evening types.

### Sleep pattern

A validated Arabic version of the ESS was used to assess the general level of daytime sleepiness for participants, which consists of four scales: normal range of sleepiness, mild, moderate, and severe sleepiness (29, 30). The ESS instrument subjectively measures general levels of sleepiness in everyday life situations. Participants rate their chances of dozing in eight situations on a 4-point scale (0 = no chance of dozing, 1 = slight chance of dozing, 2 = moderate chance of dozing, 3 = high chance of dozing) with a minimum score of 0 (normal sleep) and maximum of 24 (very sleepy).

Participants were also provided with a sleep duration diary to record their sleep times (a continuous sleep and naps ≥1 h over 24 h) for 1 week before Ramadan and another week during Ramadan.

### Physical activity

PAL was estimated using the official Arabic short-version, the self-administered format of the International Physical Activity Questionnaire (IPAQ), available online at www.ipaq.ki.se (31). IPAQ was developed to facilitate surveillance of physical activity based on a global standard, and it recalls physical activity periods of at least 10 min duration for the last 7 days (32).

IPAQ assesses physical activity undertaken across a comprehensive set of domains, including A. leisure time physical activity; B. domestic and gardening (yard) activities; C. work-related physical activity; and D. transport-related physical activity. The IPAQ short form asks about three specific types of activity undertaken in the four domains introduced above. The specific types of activity assessed are walking, moderate-intensity, and vigorous-intensity activities. The scoring protocol is applied to identify the three physical activity levels (sedentary, moderate, and vigorous). Data from the IPAQ were summarized according to the physical activities recorded (walking, moderate, and vigorous). Data were then used to estimate total weekly physical activity by weighting the reported minutes per week within each activity category by aMET energy expenditure estimate assigned to each category of activity.

### Other covariates

A self-reported questionnaire was used for sociodemographic information e.g., age, sex, marital status, education level, employment status, work time, monthly household income and smoking habits.

### Statistical analysis

Data were revised, cleaned, entered, and coded in Microsoft excel. Data analysis was then performed using SPSS version 24 (IBM, Chicago, IL, USA). Normality test was done using Kolmogorov-Smirnov test. Results are presented as frequencies and percentages for categorical variables and mean ± standard deviation (SD) or median (Q1–Q3) for continuous variables. Normally distributed variables were compared using the Student's paired *t*-test, whereas non-normal distributed variables were compared using the Wilcoxon signed-rank test. Categorical variables were compared using the Chi-square test or Fisher's Exact test (for small frequency). Statistical significance was set at *P*-value < 0.05.

## Results

**Figure 1:**
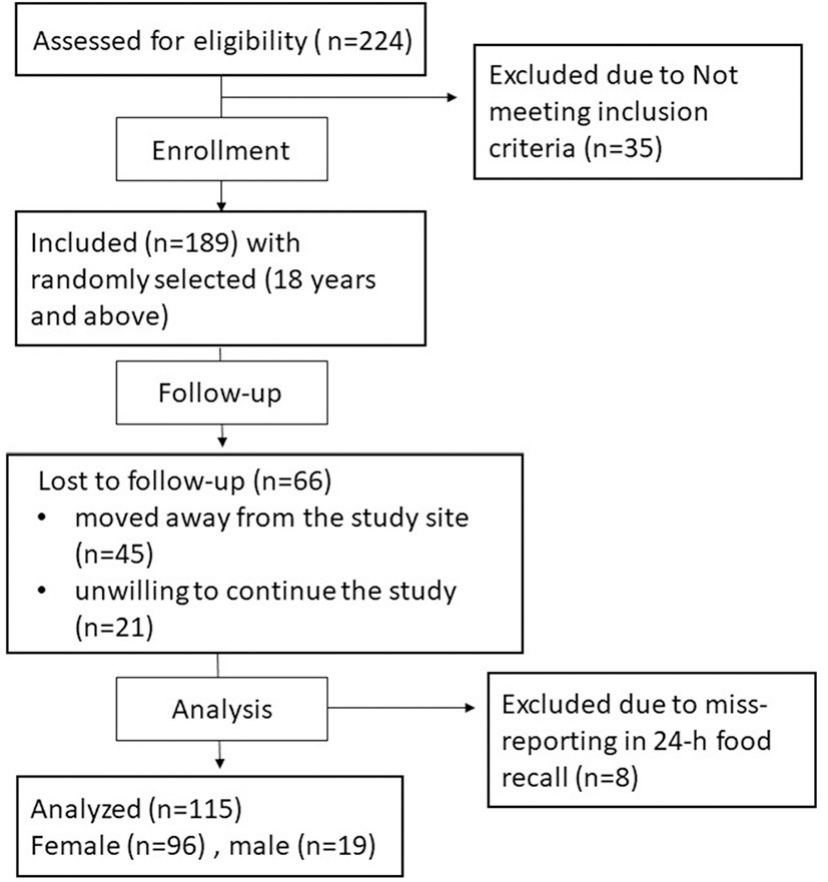
Flow chart of participants' enrollment.

Of the 224 adults who responded to the advertisements, 189 adults met the inclusion criteria. However, 66 participants withdrew before the end of the study (45 participants moved away from the study site, and 21 participants did not respond), and eight participants were excluded due to invalid reported dietary intake. Thus, a total of 115 adults (96 and 19 females and males, respectively) completed the study. The flow chart of participants' enrollment is shown in Figure 1.

## Participants characteristics

Table 1 shows the sociodemographic characteristics of study participants. A large proportion of participants were single (51.3%), had a bachelor's degree (73%), had morning jobs (73.6%), and with monthly income between SR 5,000–15,000 (54.8%).

**Table 1:** Socio-demographic characteristics for study participants (*n* = 115).

**Characteristics**	***N* (%)**
**Sex**	
Male	19 (16.5)
Female	96 (83.5)
**Marital status**	
Single	59 (51.3)
Married	49 (42.6)
Widowed	1 (0.9)
Divorced	6 (5.2)
**Qualification**	
Less than high school	5 (4.3)
High school	18 (15.6)
Bachelor's degree	84 (73.0)
Postgraduate degree	8 (7.0)
**Employment status**	
Student	18 (15.6)
Employed	72 (62.6)
Unemployed	25 (21.7)
**Working time**	
AM	53 (73.6)
PM	4 (5.5)
Shift	15 (20.8)
**Monthly income (SR)**	
<5,000	20 (19.2)
5,000–15,000	57 (54.8)
16,000–30,000	26 (25.0)
>30,000	1 (1.0)

Data are expressed as frequency and percentage *N* (%).SR, Saudi Riyal.

## Anthropometric measurements

Table 2 displays the changes in anthropometric measurements for participants before and during Ramadan. Significant decreases in body weight and BMI during Ramadan than before Ramadan were noted. On the other hand, no significant changes neither in body fat nor visceral fat were found between the two periods.

**Table 2:** Anthropometric measurements for participants before and during Ramadan (*n* = 115).

**Variables**	**Before Ramadan**	**During Ramadan**	*P*-value^*^
Body weight (kg)	66.4 ± 18.1	66.1 ± 17.8	**0.02**
BMI (kg/m^2^)	26.4 ± 6.1	26.3 ± 6.0	**0.02**
Body fat (%)	39.8 (32.2–45.6)	39.1 (33.3–45.4)	0.24
Visceral fat	6.0 (4.0–8.0)	6.0 (4.0–8.0)	0.70

Data are expressed as mean ± SD or median (Q1–Q3).

* *P*-values were derived from Student paired *t*-test and Wilcoxon signed-rank test for normal and non-normal distributed continuous variables, respectively. Bold indicates significance.

## Dietary intake

Table 3 shows the dietary intake of participants before and during Ramadan. The mean intake of total calories and carbohydrates (g/day) significantly increased during Ramadan compared with before Ramadan. On the other hand, there was a significant decrease in calorie consumption from protein (%). Calorie consumption from carbohydrates (%) and fat (%) did not show significant changes between the two periods.

**Table 3:** Dietary intake for participants before and during Ramadan (*n* = 115).

**Variables/questions**	**Before Ramadan**	**During Ramadan**	*P*-value^*^
Calorie intake (kcal/day)	1,482.9 ± 536.4	1,635.5 ± 635.1	**0.01**
CHO (%)	48.2 ± 9.1	48.1 ± 8.3	0.90
Protein (%)	16.3 ± 4.9	14.8 ± 4.6	**0.01**
Fat (%)	35.6 ± 9.2	37.1 ± 9.0	0.19
CHO (g/day)	180.8 ± 72.1	202.6 ± 88.7	**0.001**
Protein (g/day)	59.3 ± 23.0	59.1 ± 23.2	0.90
Fat (g/day)	65.4 ± 63.6	69.7 ± 32.3	0.46
**Number of meals eaten daily during the weekdays:**			
1–2 meals/day	56 (49.1)	86 (75.4)	**0.00**
3–4 meals/day	55 (48.2)	28 (24.6)	
≥5 meals/day	3 (2.6)	0 (0.0)	
**Number of meals eaten daily during the weekend:**			
1–2 meals/day	54 (48.2)	69 (61.6)	**0.01**
3–4 meals/day	53 (47.3)	43 (38.4)	
≥5 meals/day	5 (4.5)	0 (0.0)	
Number of times eating meals outside the home (restaurant) in a week:
Never	4 (3.5)	30 (26.1)	**0.00**
1–2 times a week	54 (47.4)	67 (58.2)	
3–4 times a week	39 (34.2)	10 (8.7)	
≥5 times a week	17 (14.9)	8 (7.0)	
**Number of times eating fast food per week:**			
Never	14 (12.4)	44 (38.3)	**0.00**
1–2 times a week	65 (57.5)	59 (51.3)	
3–4 times a week	25 (22.1)	7 (6.1)	
≥5 times a week	9 (8.0)	5 (4.3)	
**Do you drink coffee during the week?**			
Yes	98 (85.2)	95 (82.6)	0.59
No	17 (14.8)	20 (17.4)	
**If yes, what kind of coffee:**			
Arabic coffee	68 (48.2)	80 (57.6)	0.43
Turkish coffee	25 (17.7)	25 (18.0)	
American coffee	22 (15.6)	18 (12.9)	
French coffee	12 (8.5)	7 (5.0)	
Other	14 (10.0)	9 (6.5)	
**Number of coffee cups daily consumption:**			
1 cup/day	61 (61.0)	58 (61.0)	0.69
2–3 cups/day	29 (29.0)	26 (27.4)	
4–5 cups/day	9 (9.0)	9 (9.5)	
>5 cups/day	1 (1.0)	2 (2.1)	
**Do you drink tea during the week?**			
Yes	74 (64.3)	59 (51.3)	**0.04**
No	41 (35.7)	56 (48.7)	
**If yes, what is the number of teacups daily consumption:**			
1 cup/day	50 (67.5)	38 (64.4)	0.79
2–3 cups/day	18 (24.3)	18 (30.5)	
4–5 cups/day	3 (4.1)	1 (1.7)	
>5 cups/day	3 (4.1)	2 (3.4)	

Data are expressed as mean ± SD, or frequency and percentage *N* (%).

* *P*-values were derived from Student paired *t*-test for continuous variables and Chi-square test/Fisher's Exact test (for small frequency) for categorical variables. Bold indicates significance. CHO, carbohydrate.

There was a significant association between the number of consumed meals and the study periods. The number of meals eaten daily during weekdays and weekends decreased during Ramadan compared to before Ramadan. Associations between study periods, the number of times eating meals outside the home (restaurant) per week, and the number of times eating fast food per week were also reported.

Regarding drinking coffee, there were no significant associations between drinking coffee during the week, types of coffee and number of coffee cups consumed daily, and the study periods. On the other hand, drinking tea was significantly associated with the study periods.

## Chronotype

Table 4 details the chronotypes of study participants in the two periods. As shown in the table, an association between chronotypes and the study period was observed. Participants who had evening chronotype increased from 27.8% before Ramadan to 43.9% during Ramadan. A reduction in the percentage of morning chronotype participants during Ramadan (6.1%) compared with before Ramadan (24.3%) was also shown.

**Table 4:** Chronotypes and sleep patterns for participants before and during Ramadan (*n* = 114).

**Variables**	**Before Ramadan**	**During Ramadan**	*P*-value^*^
**MEQr**			
Evening type	32 (27.8)	50 (43.9)	**<0.001**
Morning type	28 (24.3)	7 (6.1)	
Neutral type	55 (47.8)	57 (50)	
**ESS**			
Normal sleepiness	89 (77.4)	96 (83.5)	**<0.001**
Mild sleepiness	18 (15.7)	13 (11.3)	
Moderate sleepiness	6 (5.2)	5 (4.3)	
Severe sleepiness	2 (1.7)	1 (0.9)	
**Sleep duration (hour)**	7.54 ± 1.69	7.59 ± 1.04	0.83

Data are expressed as frequency and percentage *N* (%) or mean ± SD.

* *P* values were derived from Chi-square for ESS and MEQr and paired *t*-test for sleep duration. Bold indicates significance. MEQr, Morningness-Eveningness Questionnaire; ESS, Epworth Sleepiness Scale.

### Sleep pattern

As shown in Table 4, there was a significant change in participants' ESS before and during Ramadan, with an increased percentage of participants in the normal range of sleepiness (from 0 to 10) during Ramadan compared to before Ramadan. Sleep duration did not show a significant change between the two study periods.

### Physical activity

**Figure 2:**
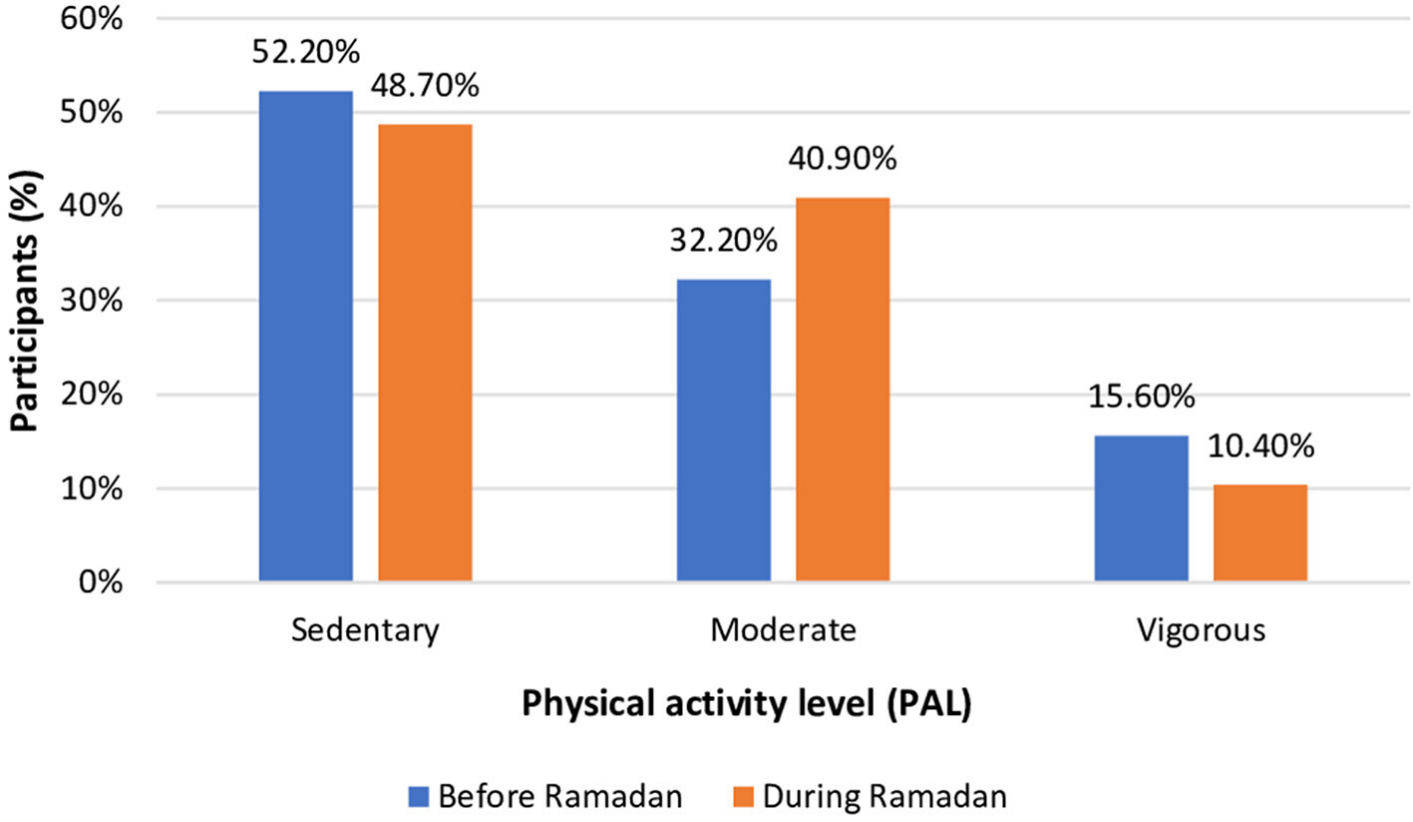
Physical activity level (PAL) for participants before and during Ramadan (*n* = 115). *P*-value = 0.19, Chi-square analysis.

PAL before and during Ramadan for the study participants is shown in Figure 2. Most participants during the two periods of the study had a sedentary level of physical activity. Overall, there was no significant difference in PAL before and during Ramadan.

### Smoking habits

A comparison of smoking habits for participants before and during Ramadan is shown in Table 5. Few participants were smokers or waterpipes consumed, and there was no significant difference in the number of cigarettes and waterpipes consumed before and during Ramadan.

**Table 5:** Smoking habits for participants before and during Ramadan (*n* = 115).

**Questions**	**Before Ramadan**	**During Ramadan**	*P*-value^*^
**Do you smoke?**			
Yes	9 (7.8)	9 (7.8)	1.00
No	106 (92.2)	106 (92.2)	
**If yes, how many cigarettes/day:**			
1–3 cigarettes/day	4 (50.0)	2 (28.6)	0.81
4–6 cigarettes/day	1 (12.5)	2 (28.6)	
>6 cigarettes/day	3 (37.5)	3 (42.8)	
**Do you smoke waterpipes?**			
Yes	6 (5.3)	8 (7.0)	0.80
No	99 (86.8)	100 (87.0)	
Sometimes	9 (7.9)	7 (6.0)	
**If yes, how many times do you smoke waterpipes/day?**			
1–2 times /day	12 (100)	14 (93.3)	1.00
3–4 times /day	0 (0.0)	1 (6.7)	

Data are expressed as frequency and percentage *N* (%).

* *P*-values were derived from Chi-square test/Fisher's Exact test (for small frequency).

## Discussion

The current study evaluated the changes in dietary intake, chronotype, sleep pattern, and physical activity in healthy Saudi adults before and during Ramadan. Our findings showed a greater mean daily calorie and carbohydrate (CHO, g/day) intake during Ramadan than before Ramadan. Moreover, there were significant associations between chronotype and sleepiness with Ramadan diurnal fasting. A significant reduction in body weight and BMI in Ramadan than before Ramadan was also noted.

Despite the restricted eating hours in Ramadan, the calorie and carbohydrate intake during Ramadan in the current study was greater than before Ramadan. Many previous studies reported an increase in total calorie intake during Ramadan (19, 33–35). However, some studies were not in agreement with our results. For example, a study reported a decrease in calorie, carbohydrate, and protein intake during Ramadan, and authors attributed that to the long fasting duration in summer when people tend to consume more fluids to cope with thirst (15). Findings from some previous studies examining the effect of Ramadan fasting on total caloric and macronutrient intake during Ramadan are listed in Table 6. Such inconsistency might be attributed to the geographical, seasonal and cultural context where the studies have been conducted. The variance in dietary intake methods used in the previous studies may also be attributed to the mixed results. Most published studies during Ramadan showed an absence of considerable changes in total daily caloric intake during Ramadan compared with the pre-Ramadan caloric intake (42–47). Nevertheless, there are significant cultural factors that may affect caloric intake during Ramadan, where some previous studies have shown that caloric intake during Ramadan decreases in Saudi Arabia (48–50). Despite the increased caloric intake, it has been shown that intermittent fasting has a beneficial effect on metabolism (51).

**Table 6:** Summarized results of the impact of Ramadan diurnal fasting on body weight, BMI, calorie intake and macronutrients.

**References**	**Participants**	**Country**	**Results**		
			**Body weight/BMI**	**Calorie intake**	**Macronutrients**
Rahman et al. (36)	20 males (healthy)	Bangladesh	Decreased in Ramadan	–	–
Sadiya et al. (37)	Nine males and 14 females (patients with metabolic syndrome	UAE	Decreased after Ramadan	–	–
Hosseini et al. (38)	22 females (healthy)	Iran	Decreased at the end of Ramadan	–	–
Savas et al. (39)	34 females (obese)	Turkey	No difference before and after Ramadan	–	–
Yeoh et al. (40)	23 males and females (patients with T2D)	Singapore	No change at the end of Ramadan	–	–
Hammoud et al. (41)	30 males and 28 females (patients with hypertension)	Lebanon	No change in females, decrease in males	–	–
Lamri-Senhadji et al. (33)	22 males and 24 females (healthy)	Algeria	–	Increased in Ramadan	High CHO, no change in protein and fat
Vasan et al. (34)	70 males and females (patients with T2D)	India	–	Increased in Ramadan	Increased in Ramadan
Shalaei et al. (35)	119 males and 147 females (healthy)	Iran	–	Increased in Ramadan	Increased in Ramadan
Eltoum et al. (19)	54 adolescents (patients with T1D)	Saudi Arabia	–	Increased in Ramadan	Increased in Ramadan
Kocaaga et al. (15)	31 males (healthy)	Turkey	–	Decreased in Ramadan	Decreased in CHO and protein, no change in fat

CHO, Carbohydrate.

It has been suggested that the changes in meal and sleep timing that occur during Ramadan might lead to a shift in circadian rhythm with the long duration of this practice (1 month), and this may be associated with changing sleep patterns during Ramadan to adapt to new meal times (52). Accordingly, a change in participants' chronotypes during Ramadan may affect dietary intake. These findings are due to the nightly restriction and practice of consuming meals at home with the family during Ramadan in Saudi society. Previous studies have indicated that high fat and sugar consumption can cause changes in clock-genes representation in the suprachiasmatic nucleus and peripheral nuclei in the brain (11, 53, 54). Moreover, other attendant lifestyle changes might affect the circadian rhythm; a previous study in Saudi Arabia demonstrated an increase in the evening chronotype among non-fasting non-Muslim residents during Ramadan, suggesting that reasons other than the timing of meals may impact the circadian rhythm during Ramadan (55).

The number of times individuals ate at restaurants or ate fast food decreased during Ramadan. These findings could be explained by the change in food preferences when people fast compared with before Ramadan.

In the current study, the mean daily sleep duration among participants was seven to 8 h during the two study periods, which is in the range of recommended sleep duration for overall health (7–9 h for adults) (56, 57). We did not find significant variances in sleep duration before and during Ramadan, and this finding agrees with a previous study that did not report a significant change in total sleep time before, during, or after Ramadan (58). However, several studies contradict our results, as they reported a decrease in total sleep duration in Ramadan (59–61). People abstain from eating and drinking in the daytime during Ramadan and change their mealtimes to be exclusively nocturnal; they also get up early before sunrise to eat Suhur or stay awake. The practice of other worship, including Taraweeh prayers during the night, is a possible explanation for decreased sleep during Ramadan noted in the previous studies (15, 61).

In this study, there was a significant association in ESS before and during Ramadan among study participants. In contrast, a study reported no significant change in the ESS during or after Ramadan, knowing that participants had a control for caloric intake, food composition, bedtime, and wake-up time. (58).

Physical activity is a part of individuals' lifestyles that may change during Ramadan. In the present study, there was no association between PAL and the study periods, These results concur with a previous study, wherein no significant change in PAL during Ramadan compared with before Ramadan was reported (18, 62). Another study reported a high prevalence of low physical activity among individuals with type 2 diabetes with no changes between during and after Ramadan (61).

The effects of Ramadan diurnal fasting on body weight have been questioned, and many studies have been conducted to explore these effects. A recent systematic review on the impact of Ramadan on body weight among healthy adults demonstrated that Ramadan diurnal fasting leads to a significant but small reduction in body weight (63). More results of published literature about the impacts of Ramadan diurnal fasting on body weight and BMI are presented in Table 6, and some contradictory results were observed. Changes in body weight due to Ramadan diurnal fasting are variable and depend on total calorie intake during Ramadan compared with before Ramadan (64). It is well-known that negative energy balance results in weight loss. Interestingly, body weight and BMI findings in this study were incompatible with neither caloric intake nor PAL findings. Other factors, such as changes in meal timing and chronotype rather than negative energy balance, might be the main driver of weight loss observed in this study. Manipulation of meal timing (i.e., Ramadan diurnal fasting) has been proposed as a dietary strategy that promotes weight loss (65). The main premise of fasting is to enhance changes in metabolic pathways, hormonal secretions, and cellular processes (66). Further research into the role played by circadian rhythm in human weight regulation is inevitable.

Another explanation of the inconsistency of body weight and energy intake findings is that the data generated by using a single 24-h recall may not represent the habitual diet of the participants. Therefore, repeat 24-h recalls are recommended in further research to capture the variation that is normally present in diet.

There are some limitations in the present study that need to be addressed. First, our findings are based on only 115 adults from Jeddah city, and the majority of them were women, which means they are not representative of the entire Saudi population. Nevertheless, most previous studies from the region were conducted on men; therefore, studies on the effect of Ramadan fasting on sleep among women are needed. Thus, larger cohort studies that include a large sample size (men and women) from different regions of Saudi Arabia are warranted. Second, despite the use of proven, reliable, and international questionnaires, which are widely used in research sitting, these remain the approaches of subjective assessment of the chronotype, sleep, and physical activity variables and might be subject to bias. Third, sleep quality was not assessed in this study. Finally, the disadvantage of the 24-h recall method includes the inability of a single day's intake to capture the habitual diet, and as a retrospective method, it relies on subject recall.

## Conclusion

This study showed that Ramadan diurnal fasting was associated with changes in dietary intake, including greater daily energy and total CHO intake as well as changes in chronotype and daytime sleepiness with no changes in sleep duration or PAL. The study also suggests that the Ramadan fasting model may be a promising weight loss strategy. However, future work concerning the lifestyle changes in Ramadan in different Islamic cultures is needed.

## Data availability statement

The original contributions presented in the study are included in the article/supplementary material, further inquiries can be directed to the corresponding author/s.

## Ethics statement

The studies involving human participants were reviewed and approved by Institutional Review Board (IRB), College of Medicine, King Saud University (Approval No. E-19-3706). The patients/participants provided their written informed consent to participate in this study.

## Author contributions

AA, MA, and AB: study concept and design and findings interpretation. AA: data collection and writing—original draft preparation. AA and MA: statistical analysis. MA and AB: writing—review and editing. All authors critically read and approved the final draft.

## Funding

Researchers Supporting Project Number (RSP-2021/338), King Saud University, Riyadh, Saudi Arabia.

## Conflict of interest

The authors declare that the research was conducted in the absence of any commercial or financial relationships that could be construed as a potential conflict of interest.

## Publisher's note

All claims expressed in this article are solely those of the authors and do not necessarily represent those of their affiliated organizations, or those of the publisher, the editors and the reviewers. Any product that may be evaluated in this article, or claim that may be made by its manufacturer, is not guaranteed or endorsed by the publisher.
